# Fellowships, Grants, & Awards

**Published:** 2004-11

## Tools for Genetic and Genomic Studies in Emerging Model Organisms

This Program Announcement (PA) is to encourage investigator-initiated applications for research designed to generate genetic tools and genomic resources that will enable researchers to exploit the full potential of novel or developing model systems for comparative and functional genomic studies. The typical model organism to be considered should have a publicly available draft of the genomic DNA sequence with a minimum of 5X coverage. In addition, it should have at least one of the following characteristics: shows promise as, or is, a model for basic biological or behavioral mechanisms; occupies an important evolutionary niche that may yield novel insights in comparative studies; or has potential as, or is, a model for developmental or disease processes. Applicants are expected to ensure that reagents, technologies, and resources developed under this initiative are made widely available to the research community.

This PA is not intended to encourage genome sequencing projects or studies of model organisms for which there are well-established databases and other genome-related resources, e.g, mouse, *Drosophila, C. elegans,* and *S. cerevisiae*. In general, resources to study organisms that fit primarily within the mission of another National Institutes of Health (NIH) Institute or Center, such as pathogenic microorganisms, are not encouraged through this PA.

The advent of the genomic era has been a boon for the investigation of a growing number of model organisms. Completion of DNA sequencing of each genome presents opportunities for novel insights into genomic function, the regulation of gene expression, and evolutionary processes. Yet, the large scale of many sequencing projects and the sheer volume of sequence data create a considerable challenge for the individual investigator as well as consortia of researchers to obtain the resources and tools required to make maximal use of genomic information for comparative or functional studies.

The major goal of this PA is to support research that will enhance the usefulness of DNA sequence information for newly emerging or developing model organisms for which there are limited genomic resources. Objectives to be addressed in applications submitted in response to this PA include, but are not limited to, the following: 1) improvements in tools for mining of data for genomes having unique composition or structure; 2) improved database management and integration with other databases (requests for the maintenance of databases alone are not encouraged); 3) generation of comprehensive cDNA libraries; 4) development of microarray reagents and/or services; 5) improved methods for linking expression arrays with standard phenotypes or with specific biological or behavioral outcomes; 6) development of novel approaches for mutagenesis and for rapid identification and characterization of point mutations; 7) development of novel transposable element-based techniques for the generation of knockouts or other mutations; 8)improvements in gene transfer technology and in vectors for genomic manipulation; and 9) generation of sets of gene knockouts or knock-downs.

This PA will use the NIH research resource grant (R24) mechanism. Responsibility for the planning, direction, and execution of the proposed project will be solely that of the applicant. The total project period for an application submitted in response to this PA may not exceed four years. A maximum of $250,000 direct costs (exclusive of subcontractual indirect costs) per year will be provided.

This PA uses just-in-time concepts. It uses the non-modular budgeting format. Follow the instructions for non-modular budget research grant applications. This program does not require cost sharing as defined in the current NIH Grants Policy Statement at http://grants.nih.gov/grants/policy/nihgps_2003/NIHGPS_Part2.htm.

The NIH is interested in ensuring that the research resources (constructs, reagents, cell lines, software tools, expression data, methods, etc.) developed through this PA become readily available to the research community for further research, development, and application, in the expectation that this will lead to products and knowledge of benefit to the public. At the same time, NIH recognizes the rights of grantees to elect and retain title to subject inventions developed under federal funding under the provision of the Bayh-Dole Act.

This PA has two special requirements regarding research resources produced in proposed projects: 1) Applicants are required to include in their applications a specific plan by which they will share research resources with the wider scientific community. A reasonable time frame for periodic deposition of mutants, reagents, and data should be specified in the application. 2) Applicants are required to include a plan addressing if, or how, they will exercise their intellectual property rights while making available to the broader scientific community patentable research resources. The plan should address the following questions: Will material transfers be made with no more restrictive terms than in the Simple Letter Material Transfer Agreement or the Uniform Biological Material Transfer Agreement? Will there be reach-through requirements on materials transferred? Should any intellectual property arise that requires a patent, will the technology remain widely available to the research community?

Both the sharing and intellectual property plans should, at a minimum, address these elements in a clear and concise manner. Applicants are encouraged to inform and/or confer with their institutional offices of technology transfer to develop plans for addressing these requirements.

Applicants are reminded that the grantee institution is required to disclose each subject invention to NIH within two months after the inventor discloses it in writing to grantee institutional personnel responsible for patent matters. The awarding Institute reserves the right to monitor awardee activity in this area to ascertain if patents or patent applications are adversely affecting the goals of this PA.

Applications must be prepared using the PHS 398 research grant application instructions and forms (rev. 5/2001). Applications must have a Dun and Bradstreet (D&B) Data Universal Numbering System (DUNS) number as the Universal Identifier when applying for federal grants or cooperative agreements. The DUNS number can be obtained by calling 866-705-5711 or through the web site at http://www.dunandbradstreet.com/. The DUNS number should be entered on line 11 of the face page of the PHS 398 form. The PHS 398 is available at http://grants.nih.gov/grants/funding/phs398/phs398.html in an interactive format. For further assistance contact GrantsInfo, 301-435-0714, e-mail: GrantsInfo@nih.gov. The title and number of this PA must be typed on line two of the face page of the application form and the YES box must be checked.

In the background section, the applicant should include a description of existing publicly available resources for the model organism being studied. The applicant should define how the proposal will enhance available resources and provide evidence of research community consultation and consensus regarding the potential value of the resource. In a brief section following the research plan, the applicant must describe plans to share research resources and to exercise intellectual property rights

Applications submitted in response to this PA will be accepted at the standard application deadlines, which are available at http://grants.nih.gov/grants/dates.htm. Application deadlines are also indicated in the PHS 398 application kit.

Submit a signed, typewritten original of the application, including the checklist, and five signed photocopies in one package to: Center for Scientific Review (CSR), NIH, 6701 Rockledge Drive, Room 1040, MSC 7710, Bethesda, MD 20892-7710 USA; Bethesda, MD 20817 (for express/courier service).

Applications must be mailed on or before the receipt dates described at http://grants.nih.gov/grants/funding/submissionschedule.htm. The CSR will not accept any application in response to this PA that is essentially the same as one currently pending initial review unless the applicant withdraws the pending application. The CSR will not accept any application that is essentially the same as one already reviewed. This does not preclude the submission of a substantial revision of an unfunded version of an application already reviewed, but such application must include an introduction addressing the previous critique.

Contact: Anthony Carter, Division of Genetics and Developmental Biology, National Institute of General Medical Sciences (NIGMS), Building 45, Room 2AS-25R, MSC 6200, Bethesda, MD 20892-6200 USA, 301-594-0943, fax: 301-480-2228, e-mail: CarterA@nigms.nih.gov.

Reference: PA No. PA-04-141

## NLM Research Grants in Biomedical Informatics and Bioinformatics (R01)

The purpose of this PA is to reissue and update the National Library of Medicine’s (NLM) research grant program for biomedical informatics and bioinformatics. NLM’s research funding centers on understanding data, information and knowledge, in particular their nature, forms and uses in the domains of health care and basic biological sciences.

NLM defines biomedical informatics as the intersection of basic informational and computing sciences with an application domain in biomedicine, as discussed in the work of the American College of Medical Informatics referenced below. The term biomedical informatics encompasses the closely-aligned field of bioinformatics, which can be defined as the intersection of basic informational and computer sciences with an application domain in biological/biochemical sciences. NLM’s research focuses on management and efficient utilization of data, information, and knowledge in health care and basic biomedical sciences.

In clinical medicine, health services administration, education, and basic biomedical sciences, computers and networks are fundamental tools of discovery, learning, decision making and management. NLM’s biomedical informatics research grants support the study of how information is best captured, represented, stored, retrieved, manipulated, managed and disseminated for use in these kinds of activities.

The following general themes demonstrate the range and scope of NLM’s research interests in biomedical informatics and bioinformatics: 1) information and knowledge processing, including natural language processing, information extraction, integration of data from heterogeneous sources or domains, event detection, feature recognition; 2) tools for analyzing and/or storing very large datasets, including genomic and proteomic data, data supporting clinical trials, and other data used in clinical or health services research; 3) knowledge representation, including vocabularies, ontologies, simulations and virtual reality; 4) linkage of clinical and genomic information to benefit health care; 5) innovative uses of information technology in health care delivery, including decision support, error reduction, outcomes analysis, and information at the point of care; 6) efficient management and utilization of information and data, including knowledge acquisition and management, process modeling, data mining, acquisition and dissemination, novel visual presentations, and stewardship of large-scale data repositories and archives; 7) human-machine interaction, including interface design, use and understanding of health related-information, intelligent agents, information needs and uses. 8) high-performance computing and communications relating to biomedical applications, including efficient machine-machine interfaces, transmission and storage, and real-time decision support; 9) innovative uses of information technology to enhance learning, retention and understanding of health-related information.

Informatics research is interdisciplinary and employs a range of research methodologies. NLM expects that investigators will employ sound techniques that lead to the collection and analysis of empirical evidence. These techniques may include quantitative and/or qualitative approaches, including laboratory and field studies, surveys and needs analyses, ’in silico’ experiments, modeling and simulation studies.

NLM is a participant in the National Institutes of Health (NIH) Roadmap initiatives, many of which include biomedical computing and interdisciplinary research as essential elements, in the programs of the NIH BISTI initiative, and other NIH informatics initiatives.

While biomedical informatics research projects funded by this program often require software development and tool-building, a well-defined research problem and rigorous research design are essential elements of NLM’s R01 grants. Investigators interested in demonstration projects, proofs of concept or other feasibility testing should consider NLM’s Exploratory/Developmental grant program (http://grants.nih.gov/grants/guide/pa-files/PA-03-107.html) rather than this biomedical informatics research grant program. NLM’s Small Project grants (http://grants.nih.gov/grants/guide/pa-files/PA-03-108.html) are most appropriate for investigators who are just beginning their research in an area and/or need preliminary data to inform a more substantial research project.

Research in biomedical informatics or bioinformatics often employs a specific scientific discipline or medical subspecialty as the subject field or domain in which the research is undertaken, or in which tools and ideas are applied. However, grant applications whose primary focus is on a disease or biological question, rather than the informatics or computational issues that pertain to them, are more appropriate for other Institutes at NIH.

This PA will use the NIH R01 award mechanism. As an applicant, you will be solely responsible for planning, directing, and executing the proposed project.

This PA uses just-in-time concepts. It also uses the modular budgeting as well as the non-modular budgeting formats (see http://grants.nih.gov/grants/funding/modular/modular.htm). Specifically, if you are submitting an application with direct costs in each year of $250,000 or less, use the modular budget format. Otherwise follow the instructions for non-modular budget research grant applications. This program does not require cost sharing as defined in the current NIH Grants Policy Statement at http://grants.nih.gov/grants/policy/nihgps_2003/NIHGPS_Part2.htm.

An applicant can request funding for up to five years of support. The average duration of recent NLM informatics research awards is about three years.

Applications must be prepared using the PHS 398 research grant application instructions and forms (rev. 5/2001). Applications must have a Dun and Bradstreet (D&B) Data Universal Numbering System (DUNS) number as the Universal Identifier when applying for federal grants or cooperative agreements. The D&B number can be obtained by calling 866-705-5711 or through the web site at http://www.dunandbradstreet.com/. The D&B number should be entered on line 11 of the face page of the PHS 398 form. The PHS 398 is available at http://grants.nih.gov/grants/funding/phs398/phs398.html in an interactive format. For further assistance contact GrantsInfo, 301-435-0714, e-mail: GrantsInfo@ nih.gov. The title and number of this PA must be typed on line two of the face page of the application form and the YES box must be checked.

Applications submitted in response to this PA will be accepted at the standard application deadlines, which are available at http://grants.nih.gov/grants/dates.htm. Application deadlines are also indicated in the PHS 398 application kit.

Submit a signed, typewritten original of the application, including the checklist, and five signed photocopies in one package to: Center for Scientific Review, NIH, 6701 Rockledge Drive, Room 1040, MSC 7710, Bethesda, MD 20892-7710 USA; Bethesda, MD 20817 (for express/courier service).

Applications must be mailed on or before the receipt dates described at http://grants.nih.gov/grants/funding/submissionschedule.htm. The CSR will not accept any application in response to this PA that is essentially the same as one currently pending initial review unless the applicant withdraws the pending application. The CSR will not accept any application that is essentially the same as one already reviewed. This does not preclude the submission of a substantial revision of an unfunded version of an application already reviewed, but such application must include an introduction addressing the previous critique.

Contact: Valerie Florance, Deputy Director, Extramural Programs Division, National Library of Medicine, Rockledge 1, Suite 301, 6705 Rockledge Drive, Bethesda, MD 20892-0001 USA, 301-594-4882, fax: 301-402-2952, e-mail: floranv@ mail.nih.gov; Hua-Chuan Sim, Scientific Review Administrator, Extramural Programs Division, National Library of Medicine, Rockledge 1, Suite 301, 6705 Rockledge Drive , Bethesda, MD 20892-0001 USA, 301-496-4253, fax: 301-402-2952, e-mail: simh@mail.nih.gov

Reference: PA No. 04-141

## Quick-Trials for Novel Cancer Therapies: Exploratory Grants

Continuing progress in basic cancer research and drug development has led to discoveries of new agents or approaches for molecular targeting in novel cancer therapies. These new agents or approaches suppress tumor growth through multiple mechanisms such as cell cycle control, activation of tumor suppressor genes, essential signal pathway blockage, tumor vaccines, tumor microenvironment modification, etc. Rapid translation of these exciting discoveries into clinical practice requires timely support to accommodate the special needs of novel cancer therapy development. This Program Announcement (PA) is intended to provide investigators with rapid access to support for pilot, Phase I, and Phase II cancer clinical trials as well as support for patient monitoring and laboratory studies linked to a cancer clinical trial. Phase III trials are not excluded but such trials generally require greater resources and duration than available from an R21 award. Applications that do not propose a cancer clinical trial or patient monitoring or laboratory studies linked to a cancer clinical trial may be returned to applicants without being reviewed. The focus of this Quick-Trial PA is on translational research in new agent development to ensure the timely exploitation of new cancer therapeutic approaches including the development of new cancer prevention agents. This PA is aimed at providing a new approach in the grant application process by offering a rapid turnaround from application submission to funding. Features of this initiative include a modular grant application and award process, inclusion of the clinical protocol within the grant application, and an accelerated peer review with the goal of issuing new awards within six months of application receipt. Inclusion of the complete clinical protocol within the PHS 398 grant application is intended to simplify the application process by eliminating the need to duplicate protocol details in the Research Plan section and to insure proper peer review of the application. In addition, Quick-Trial applications do not require extensive preliminary data in the grant application and support exploratory translational and clinical research studies involving cancer prevention, chemotherapy and rapid development and application of novel clinical cancer therapies including image guided therapeutic procedures. Investigators may apply for a maximum of two years of funding support using the exploratory or developmental (R21) grant mechanism for up to $250,000 direct costs per year.

Advances in the understanding of molecular cancer genetics, basic cancer biology, and the development of powerful technologies such as microarray, proteomics and bioinformatics have led to the identification of many new molecular targets and pathways in cancer cells. These discoveries have created new frontiers for novel molecular cancer prevention and treatment leading to the development of molecular medicine in cancer therapy. In addition, these novel targets and pathways have presented excellent translational research opportunities for revolutionizing cancer drug development and bringing more effective molecular cancer therapies and cancer prevention strategies into clinical practice.

Novel approaches or agents for inhibiting tumor growth either directly or by impacting the tumor microenvironment are ready to be tested in the clinic with new tools and laboratory analyses that allow investigators to ascertain how specific targets are affected by therapy. These agents include new classes of cytotoxic agents, agents or approaches that act via immune-stimulatory effects, agents that stimulate apoptosis, inhibit angiogenesis and metastasis or alter tumor cell signaling pathways, and agents targeted specifically to novel cancer cell targets. New clinical therapeutic trials may employ drugs/agents, biologics, radiation, heat, or surgery used as single agents/modalities or in combination for the treatment of early and advanced disease. In addition, clinical trials of therapies for cancer treatment, including but not limited to herbal therapies, dietary supplements, bioactive food components, or unconventional pharmacological and biological interventions (e.g. antineoplastons, Coley's toxin, enzyme therapies, etc.) will be considered. Another relevant area of investigation is the use of anatomical and molecular image guidance for targeted treatment with ablative techniques or delivery of chemotherapeutic agents. At present, there are few funding mechanisms targeted to stimulate the translation of promising and potentially relevant advances in new prevention or therapeutic agent development from the laboratory into the clinical setting. Quite frequently the initial stages of clinical investigation are the most difficult to accomplish. They are resource intensive, and, to be done well, they require laboratory, pharmacology, and other resource support, as well as substantial personnel effort, none of which is supported by traditional health benefit programs. Nonetheless, these early studies tend to fare poorly in competition for conventional grant support precisely because they are preliminary and cannot serve as the definitive tests of new approaches. Even when funding is received, the review and award cycle may introduce a year or more of delay. Except where there is an industrial sponsor with a particular commitment to development of an agent, it may take a long time for a promising approach to get through the initial phase of demonstrating feasibility and interest, or it may never be tested in more than one or two diseases.

This PA will continue to support scientific, technological, clinical and logistical needs in novel cancer therapy development. In addition, this PA will complement the Rapid Access to Intervention Development (RAID) program http://dtp.nci.nih.gov/docs/raid/raid_index.html by providing an initiative with accelerated peer review and funding to support the clinical and laboratory costs of early clinical testing to ensure the timely development of new therapeutic approaches.

This PA will use the NIH exploratory/development (R21) award mechanism. As an applicant, you will be solely responsible for planning, directing, and executing the proposed project. The applicant may request a project period of up to 2 years with direct costs limited to $250,000 per year.

This PA uses just-in-time concepts. It also uses the modular budgeting format (see http://grants.nih.gov/grants/funding/modular/modular.htm). This program does not require cost sharing as defined in the current NIH Grants Policy Statement at http://grants.nih.gov/grants/policy/nihgps_2003/NIHGPS_Part2.htm.

Applications must be prepared using the PHS 398 research grant application instructions and forms (rev. 5/2001). Applications must have a Dun and Bradstreet (D&B) Data Universal Numbering System (DUNS) number as the Universal Identifier when applying for federal grants or cooperative agreements. The D&B number can be obtained by calling 866-705-5711 or through the web site at http://www.dunandbradstreet.com/. The D&B number should be entered on line 11 of the face page of the PHS 398 form. The PHS 398 document is available at http://grants.nih.gov/grants/funding/phs398/phs398.html in an interactive format. For further assistance, contact GrantsInfo, 301-435-0714, e-mail: GrantsInfo@nih.gov. The title and number of this PA must be typed on line 2 of the face page of the application form and the YES box must be checked.

Because Quick-Trial applications will propose cancer clinical trials or patient monitoring or laboratory studies linked to a cancer clinical trial, applicants are reminded to properly complete item e (Humans Subjects Research) of the Research Plan. This is described in PHS 398. As applicable, the Human Subjects Research portion of the Research Plan includes, but is not limited to, a Data and Safety Monitoring Plan, Women, Minority, and Children Inclusion sections, and a Targeted/Planned Enrollment Table.

Submit a signed, typewritten original of the application, including the checklist, and five signed photocopies in one package to: Center for Scientific Review, National Institutes of Health, 6701 Rockledge Drive, Room 1040, MSC 7710, Bethesda, MD 20892-7710 USA; Bethesda, MD 20817 (for express/courier service).

The CSR will not accept any application in response to this PA that is essentially the same as one currently pending initial review unless the applicant withdraws the pending application. The CSR will not accept any application that is essentially the same as one already reviewed. This does not preclude the submission of a substantial revision of an unfunded version of an application already reviewed, but such application must include an introduction addressing the previous critique.

Contact: Roy Wu, Clinical Grants and Contracts Branch, Cancer Therapy Evaluation Program, Division of Cancer Treatment and Diagnosis, National Cancer Institute (NCI), 6130 Executive Boulevard, EPN Room 7009, Bethesda, MD 20892-7432 USA; Rockville, MD 20852 (for express/courier service), 301-496-8866, fax: 301-480-4663, e-mail: wur@ctep.nci.nih.gov

Reference: PA No. PAR-04-155

## Genetics and Pathobiology of Vascular Cognitive Impairment

The purpose of this Program Announcement with set-aside funds (PAS) is to invite applications to study the biological basis of vascular cognitive impairment (VCI). VCI causes a burden of illness similar to that caused by Alzheimer’s disease (AD), but has been far less well-studied. Recently, however, some important strides have been made in understanding the etiology of VCI. These include the discovery of a monogenic form of vascular dementia, CADASIL, and identification of the causative gene as Notch 3. In addition, MRI and other pathological data have provided a clearer delineation of the various clinical subtypes of VCI, and awareness of the synergistic interaction between vascular and classical Alzheimer’s pathologies in producing cognitive impairment. The goal of this PAS is to build on these first critical achievements to obtain a better understanding of the cellular and molecular mechanisms causing vascular, neural, and glial dysfunction in human VCI and animal models of VCI.

The number of people affected by dementia in the US is expected to increase three-fold in the next 50 years, to a total of over 13 million. The best-known form of dementia is AD, whose definitive diagnostic sign is the presence of plaques and tangles in brain neurons upon autopsy. However, a large proportion of dementia cases in the aged population are not due to AD, but rather to cerebrovascular disease. Dementia due to cerebrovascular disease is referred to as “vascular dementia”, and can occur in the absence of Alzheimer’s pathology. In addition to this so-called “pure” vascular dementia, there are also cases of “mixed” dementia in which cerebrovascular and Alzheimer’s pathologies coexist. Recent studies suggest that pure vascular dementia and mixed dementia together comprise the majority of dementia cases in some populations.

Vascular dementia can arise from any of several cerebrovascular disease conditions, but its two major causes are focal ischemic infarcts (i.e., strokes) and subcortical ischemic vascular disease. Focal ischemic infarcts result from occlusion of large vessels, in either cortical or subcortical locations, and are accompanied by acute clinical signs of neurological impairment. (Dementia arising from large infarcts is also sometimes referred to as “multi-infarct dementia”.) Subcortical ischemic vascular disease, on the other hand, results from occlusion of small vessels, and creates widespread small lesions (lacunae) and/or areas of demyelination. The areas affected are generally subcortical, including the basal ganglia, cerebral white matter and brainstem. This form of vascular disease generally does not produce sudden, acute symptoms, but rather causes longer-term, insidious changes in neurological function. In a significant portion of cases, this disease can even remain clinically silent for the life of the individual. Subcortical small vessel disease can be diagnosed by imaging even in cases where it is clinically silent.

In recent years, the term “vascular dementia” has been replaced by the term “vascular cognitive impairment (VCI)”. This change reflects the realization that cerebrovascular disease can cause significant cognitive and functional decline in the absence of dementia as defined by standard criteria. In addition, there is increasing evidence that VCI differs from AD in terms of precise range of cognitive defects associated with each disease. AD is characterized primarily by episodic memory loss due to loss of cholinergic basal forebrain neurons and their projections to the hippocampus. In contrast, VCI in its purest forms seems to be characterized more by loss of executive function and attentional mechanisms associated with prefrontal circuitry. However, the spectrums of defects seen in VCI and AD overlap substantially. This fact, together with the frequent coexistence of vascular and Alzheimer’s pathologies within individual patients, renders it difficult to provide definitive diagnoses based strictly on cognitive tests.

Despite the enormous prevalence of VCI, the biological basis of this disease has been much less well studied than that of AD. This lack has been due in part to the clinical heterogeneity of the disease, and also to poor understanding of its pathology at the cellular level. Recently however, research in VCI has taken some critical first steps forward. A genetic form of vascular dementia, CADASIL, has been discovered, and the mutant gene identified as Notch 3. Previous research in animal models had shown Notch 3 to be important in early neural and vascular development. The finding that mutation of Notch 3 leads to stroke and dementia (both seen in CADASIL) suggests that the gene also plays an important role in the function or maintenance of vascular and/or neural cells in the adult. Consistent with this possibility, a transgenic mouse carrying the mutant form of Notch 3 has now been generated which shows degeneration of smooth muscle cells similar to that seen in human patients. These findings provide an important foothold for understanding the cell biology as well as the genetics of VCI. Moreover, the known interaction of Notch with the presenilin proteins suggests a juncture in the disease pathways underlying VCI and AD, which also could be further explored in mouse models.

Another major area ripe for exploration concerns the genes and other risk factors that link vascular pathology to neural pathology or that render individuals susceptible to neuronal damage and cognitive impairment in response to cerebrovascular disease. Some progress has been made in recent years in defining genes that predispose individuals to stroke and cerebrovascular disease per se, but no studies have yet examined genes that control the ability of neural tissue to recover from ischemic injury. Identifying such genes would provide clear paths both to understanding the cell biology of VCI, and also to the design of protective agents and therapeutics.

Research areas appropriate for this PAS would include, but are not imited by the following examples: 1) genetics of VCI, in both animal models and humans, in particular, identification of genes that render individuals susceptible to cognitive impairment secondary to cerebrovascular disease; 2) analysis of cellular and molecular changes occurring in vascular, neuronal, and glial cells during the development of VCI in human patients, and correlation of these with MRI signs and changes in cognitive function; 3) studies of cellular and molecular pathological processes occurring in vascular, neuronal, and glial cells in animal models of VCI, such as mouse lines carrying mutant forms of Notch 3 or the stroke-prone spontaneously hypertensive rat; 4) studies of Notch 3 function in the maintenance and repair of vascular, neuronal, and glial cells in normal adult animals; studies of the cellular and molecular bases of the pathogenic actions of mutant Notch 3; 5) studies of the cellular and molecular bases of the interaction between the VCI and AD pathways (for example, studies of vascular function and pathology in animal models of AD); 6) development and characterization of new animal models for the study of VCI, and of the interaction between VCI and AD pathogenic mechanisms; 7) analysis of cognitive function in animal models of VCI, and correlation of changes in cognitive function with cellular and molecular pathologies; 8) studies on the cellular and molecular effects of hypertension, diabetes, hyperlipidemia, coagulant and anticoagulant proteins, inflammatory cytokines, and complement proteins on the vessel wall in appropriate animal models for VCI.

This PAS will use the NIH R01 and R21 award mechanism(s). As an applicant, you will be solely responsible for planning, directing, and executing the proposed project. The proposed project period during which the research will be conducted should adequately reflect the time required to accomplish the stated goals The R21 mechanism (see http://grants.nih.gov/grants/guide/pa-files/PA-03-107.html) is intended to encourage exploratory and developmental research projects by providing support for the early and conceptual stages of these projects. These one-time awards support innovative, high impact research projects that assess the feasibility of a novel area of investigation or a new experimental system, include the unique and innovative use of an existing methodology to explore a new scientific area, involve considerable risk but may lead to a breakthrough in a particular area, or develop new technology or methodology that could have major impact in a specific research area. Applications for R21 awards should describe projects distinct from those supported through the traditional R01 mechanism. For example, long-term projects, or projects designed to increase knowledge in a well-established area will not be considered for R21 awards. Applications submitted under this mechanism should be exploratory and novel. These studies should break new ground or extend previous discoveries toward new directions or applications.

R21 applications may request a project period of up to two years with a combined budget for direct costs of up to $275,000 for the two year period. For example, you may request $100,000 in the first year and $175,000 in the second year. The request should be tailored to the needs of your project. Normally, no more than $200,000 may be requested in any single year. For further information on the R21 mechanism, including Institute-specific information, see http://grants.nih.gov/grants/guide/pa-files/PA-03-107.html.

This PAS uses just-in-time concepts. It also uses the modular budgeting as well as the non-modular budgeting formats (see http://grants.nih.gov/grants/funding/modular/modular.htm). Specifically, if you are submitting an application with direct costs in each year of $250,000 or less, use the modular budget format. Otherwise follow the instructions for non-modular budget research grant applications.

The National Institute of Neurological Disorders and Stroke (NINDS) has set aside a total of $2,250,000, in addition to funds available for applications sent in response to this PA that score within the NINDS payline (see NINDS Funding Strategy http://www.ninds.nih.gov/funding/ninds_funding_strategy.htm), depending on the overall scientific merit of the applications and the availability of funds throughout the duration of this solicitation (three years). The National Institute on Aging (NIA) has set aside a total of $300,000, and the National Heart, Lung and Blood Institute (NHLBI) has set aside a total of $350,000.

PHS policy requires that investigators make unique research resources available for research purposes to qualified individuals within the scientific community when they have been published (see the NIH Grants Policy Statement at http://grants.nih.gov/grants/guide/notice-files/not96-184.html). In addition, NIH recently released a statement on the sharing of research data that applies to all investigator-initiated applications with direct costs greater than $500,000 in any single year (http://grants.nih.gov/grants/guide/notice-files/NOT-OD-03-032.html).

All applicants who respond to this PAS must propose plans for sharing data and biomaterials generated through the grant. Applicants should explain how funds for the storage and distribution of data and biomaterials will be obtained, and may request such funds in the budget of the application. It is expected that the data to be shared will be clinical, diagnostic, and pedigree structure information, and information about the genetic backgrounds and phenotypes of mutant or transgenic animal strains. Biomaterials to be shared will include patient DNAs and cell lines, and mutant or transgenic animal strains. When possible, data and biomaterials should be placed in databases or repositories that will permit their efficient distribution to investigators throughout the scientific community. An example of such a facility is the NINDS Human Genetics Resource Center (http://locus.umdnj.edu/ninds).

The Initial Review Group will evaluate the proposed sharing plan and comment on its adequacy in an administrative note in the summary statement. Reviewers will not factor the proposed data-sharing plan into the determination of scientific merit or priority score. The adequacy of the plan will be considered by NIH staff in determining whether the grant shall be awarded. The sharing plan as approved, after negotiation with the applicant when necessary, will be a condition of the award.

Our understanding of pathogenic mechanisms in VCI would benefit tremendously from the use of standardized criteria for diagnosing this condition, including standardized methods for measuring cognitive function. NINDS plans to encourage and coordinate the use of a minimal diagnostic dataset in studies funded by this PAS. Until such a dataset is defined, applicants to this PAS should provide detailed descriptions of the patient data to be collected, including methods for independently assessing the presence and type of cerebrovascular disease, and levels of cognitive function. Rationale for choice of specific cognitive test(s) should be included. In addition, plans should be included for entry of disease and cognitive phenotypic data into a computerized database that may be easily shared with other researchers.

Applicants to be funded under this PAS will be expected to travel to NIH once a year to share progress with NIH program staff, other investigators funded under this PAS, and additional advisers as deemed necessary by NIH program staff. Applicants should include funds to support one trip per year to Bethesda, MD (for the principal investigator and co-principal investigators only).

Applications must be prepared using the PHS 398 research grant application instructions and forms (rev. 5/2001). Applications must have a Dun and Bradstreet (D&B) Data Universal Numbering System (DUNS) number as the Universal Identifier when applying for federal grants or cooperative agreements. The D&B number can be obtained by calling 866-705-5711 or through the web site at http://www.dunandbradstreet.com/. The D&B number should be entered on line 11 of the face page of the PHS 398 form. The PHS 398 is available at http://grants.nih.gov/grants/funding/phs398/phs398.html in an interactive format. For further assistance contact GrantsInfo, 301-435-0714, e-mail: GrantsInfo@nih.gov. The title and number of this PA must be typed on line 2 of the face page of the application form and the YES box must be checked.

Submit a signed, typewritten original of the application, including the checklist, and five signed photocopies in one package to: Center for Scientific Review, National Institutes of Health (NIH), 6701 Rockledge Drive, Room 1040, MSC 7710, Bethesda, MD 20892-7710 USA; Bethesda, MD 20817 (for express/courier service).

Applications must be mailed on or before the receipt dates described at http://grants.nih.gov/grants/funding/submissionschedule.htm. The CSR will not accept any application in response to this PAS that is essentially the same as one currently pending initial review unless the applicant withdraws the pending application. The CSR will not accept any application that is essentially the same as one already reviewed. This does not preclude the submission of a substantial revision of an unfunded version of an application already reviewed, but such application must include an introduction addressing the previous critique.

Contact: Gabrielle G. Leblanc, Program Director, Neurogenetics, National Institute of Neurological Disorders and Stroke (NINDS), Neuroscience Center, Suite 2136, MSC 9537, Bethesda, MD 20892-0001 USA, 301-496-5745, fax: 301-402-1501; e-mail: leblancg@ninds.nih.gov; Creighton H. Phelps, Director, Alzheimer's Disease Centers Program, Neuroscience and Neuropsychology of Aging, National Institute on Aging (NIA), National Institutes of Health, 7201 Wisconsin Ave., Suite 350, Bethesda, MD, 20892-0001 USA; 301-496-9350, fax: 301-496-1494; e-mail: phelpsc@nia.nih.gov; Stephen S. Goldman, Vascular Biology Research Program, Division of Heart and Vascular Diseases, National Heart, Lung, and Blood Institute (NHLBI), 6701 Rockledge Drive, Suite 10192, MSC 7956, Bethesda, MD 20892-0001 USA, 301-435-0560, fax: 301-480-2858, e-mail: goldmans@nhlbi.nih.gov.

Reference: PA No. PAS-04-149

